# Hot in Cold: Microbial Life in the Hottest Springs in Permafrost

**DOI:** 10.3390/microorganisms8091308

**Published:** 2020-08-27

**Authors:** Tatiana V. Kochetkova, Stepan V. Toshchakov, Kseniya S. Zayulina, Alexander G. Elcheninov, Daria G. Zavarzina, Vasiliy Yu. Lavrushin, Elizaveta A. Bonch-Osmolovskaya, Ilya V. Kublanov

**Affiliations:** 1 Winogradsky Institute of Microbiology, Research Center of Biotechnology of the Russian Academy of Sciences, Moscow 117312, Russia; stepan.toshchakov@gmail.com (S.V.T.); zauylinakc@gmail.com (K.S.Z.); elcheninov.ag@gmail.com (A.G.E.); zavarzinatwo@mail.ru (D.G.Z.); elizaveta.bo@gmail.com (E.A.B.-O.); kublanov.ilya@gmail.com (I.V.K.); 2 Kurchatov Center for Genome Research, National Research Center “Kurchatov Institute”, Moscow 123182, Russia; 3 Geological Institute, Russian Academy of Sciences, Moscow 119017, Russia; v_lavrushin@ginras.ru; 4 Faculty of Biology, Lomonosov State University, Moscow 119234, Russia

**Keywords:** Chukotka, thermophiles, hot spring, NGS sequencing, microbial diversity, permafrost, polar environments

## Abstract

Chukotka is an arctic region located in the continuous permafrost zone, but thermal springs are abundant there. In this study, for the first time, the microbial communities of the Chukotka hot springs (CHS) biofilms and sediments with temperatures 54–94 °C were investigated and analyzed by NGS sequencing of 16S rRNA gene amplicons. In microbial mats (54–75 °C), phototrophic bacteria of genus *Chloroflexus* dominated (up to 89% of all prokaryotes), while *Aquificae* were the most numerous at higher temperatures in Fe-rich sediments and filamentous “streamers” (up to 92%). The electron donors typical for *Aquificae*, such as H_2_S and H_2_, are absent or present only in trace amounts, and the prevalence of *Aquificae* might be connected with their ability to oxidize the ferrous iron present in CHS sediments. *Armatimonadetes*, *Proteobacteria*, *Deinococcus-Thermus*, *Dictyoglomi*, and *Thermotogae,* as well as uncultured bacteria (candidate divisions Oct-Spa1-106, GAL15, and OPB56), were numerous, and *Cyanobacteria* were present in low numbers. Archaea (less than 8% of the total community of each tested spring) belonged to *Bathyarchaeota*, *Aigarchaeota*, and *Thaumarchaeota*. The geographical location and the predominantly autotrophic microbial community, built on mechanisms other than the sulfur cycle-based ones, make CHS a special and unique terrestrial geothermal ecosystem.

## 1. Introduction

**Figure 1:**
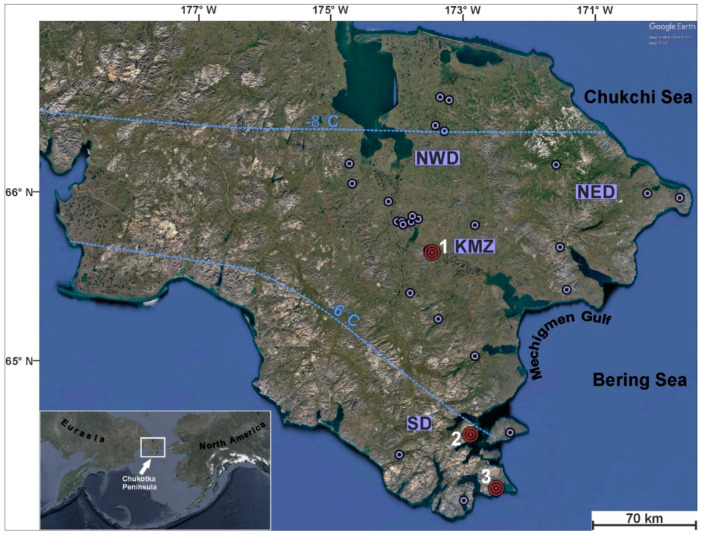
Location of hot spring groups found in the Chukotka Peninsula. Numbers 1, 2, and 3 indicate the Mechigmen, the Senyavin, and the Chaplino thermal groups, respectively. Isolines mean annual air temperatures. Different areas are shown as abbreviations SD (southern), NED (north-eastern), NWD (north-western), and KMZ (Kolyuchin–Mechigmen Zone).

Permafrost covers about 25% of the Northern Hemisphere’s terrestrial surface [1]. The Chukotka Peninsula, located in the northeast of Russia, is a typical arctic area with a continuous distribution of permafrost and is regarded as one of the coldest places on Earth, with an average temperature of −25 °C in January and +9 °C in July. It is a region with an area of approximately 60,000 km^2^, washed by the Chukchi and Bering Seas, and separated from Alaska by the Bering Strait (Figure 1). Though there are no manifestations of modern volcanism, such as active volcanoes, fumaroles, or geysers, geothermal springs with water temperatures from 4 to 97 °C are quite abundant, primarily in the northeastern part [2,3]. Chukotka hot springs (CHS) formed as a result of young (Neogene-Quaternary period, 2.6–0.1 Ma) tectonic–magmatic activity and, associated with the zone of local depression stretches from the Kolyuchin Bay to the Mechigmen Gulf, were thus named the Kolyuchin–Mechigmen Zone (KMZ) or Mechigmen Rift Zone.

The geochemical characteristics and the gas composition of CHS were analyzed in detail previously [2,3]. CHS fluids belong to chloride-carbonate types that are poor in SO_4_^2^^−^ ion concentration (0.5–2 mM); the cations Na^+^ and Ca^2+^ occur in relatively high concentrations (10–300 mM and 1–200 mM, respectively). The evolving gases of CHS are enriched in N_2_ and/or CO_2_, with CH_4_, H_2,_ and CO being the minor components, not exceeding 1.5, 0.01, and 0.0008 vol.%, respectively. The pH of CHS is near neutral to slightly alkaline (6.0–8.5) [3]. The springs differ in total dissolved salt (TDS) values (1.5–37 g/L), which can be explained by the penetration of the hot fluid through the highly mineralized water of cryopegs (lenses of natural saltwater with negative temperatures located in permafrost) or by the impact of deep circulating groundwater formed because of ancient seawater metamorphism [3,4,5,6]. As in other nonvolcanic hydrothermal systems, the hot water of CHS is discharging due to the geothermal activity, which is attributed to the tectonically driven dilation of fault systems acting as a conduit for fluids, and the subsequent heating of meteoric water (the cooling magma model and the thermal gradient model) [3,4,5,6]. This explains the absence of reduced sulfur compounds in the thermal fluids of CHS that are typical for geothermal springs discharging in active volcanic regions, such as Kamchatka (Russia) or Yellowstone National Park (USA). The springs are located at nearly 66° N and experience distinct seasonal illumination with the continuous sun during the Arctic summer and continuous darkness during the polar nights in the winter.

Hydrothermal springs located in permafrost are not a unique phenomenon, though they are rather fascinating. The most northern among currently known terrestrial geothermal springs are located in Canada [7,8] and Norway (Svalbard Island) [9] permafrost areas at a latitude of almost 80° N. Water temperatures there do not exceed 7 and 26 °C, respectively. Springs in Greenland (Denmark) are heated to 55–62 °C [10]. A few geothermal sites with temperatures ranging from 50 to 95 °C were found in the Southern Hemisphere in Antarctica [11,12]. At the same time, the temperature of water in the hottest CHS reaches 94–97 °C [2,3], making CHS, to our knowledge, the hottest documented geothermal springs in the Northern Hemisphere permafrost zone.

Data on microbial life in geothermal springs discharging in permafrost zones are scarce due to their difficult accessibility. Few previous works focused on cyanobacterial mats and the diversity of phototrophic organisms [9,10,13] in hydrothermal springs with temperatures below 62 °C, where the illumination plays a crucial role in the establishment of microbial communities. Studies of the prokaryotic diversity of Antarctic hot environments revealed the presence of *Geobacillus*, *Bacillus*, *Brevibacillus*, *Thermus*, *Thermococcus,* and *Pyrococcus* members [12].

CHS have been known since the first expeditions to the Chukotka Peninsula in the 18th century [14]; however, their microbial diversity was never explored. The goal of our work was to estimate the dominating prokaryotes in CHS with different temperatures, the chemical composition of water, and the mineral composition of the sediments. Here, we describe the results of the first CHS molecular ecology studies of the biofilms and sediments that were collected from hot springs in the northeastern part of the Chukotka Peninsula (Figure 1).

## 2. Materials and Methods 

### 2.1. Characteristics of the Sampling Sites

During the expedition to Chukotka in July–August 2016, samples of sediments, microbial mats, and filamentous “streamer” communities were collected from the hot springs of three thermal groups (Figure 1): Mechigmen (65°48′ N, 173°24′ W), Senyavin (64°44′ N, 172°51′ W), and Chaplino (64°25′ N, 172°30′ W). 

**Figure 2:**
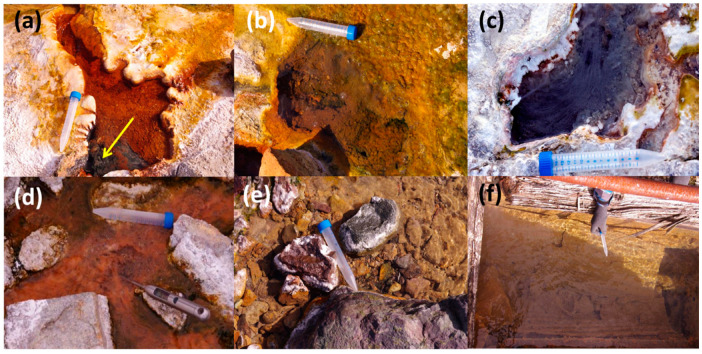
Photographs of Chukotka hot springs: (**a**) spring 3730 (94 °C/pH 6.9/Eh −300) with a yellow arrow, indicating “black sediments”, (**b**) spring 3723 (67 °C/pH 6.7/Eh −70), and (**c**) spring 3717 (69 °C/pH 6.8/Eh −326) of the Mechigmen group; (**d**) spring 3735 of the Senyavin group (60 °C/pH 8.0/Eh +220); (**e**) spring 3751 (75 °C/pH 7.5/Eh +30/+80) in the “Upper”, and (**f**) spring 3756 (67 °C/pH 7.3/Eh +10) in the “Lower” groups of the Chaplino thermal field.

Most of the samples were taken from the Mechigmen Spring Group, the largest and most active in Chukotka (in terms of water discharge), located in the KMZ, 65 km to the northwest of Mechigmen Bay. The group is located in a hard-to-reach and uninhabited area in a floodplain of the Igelchveem River. The valley follows a powerful fault zone with porphyry, granite-porphyry, and tuffs on its sides. The thermal field stretches for 850 m along both banks of the river. The main evolving gases in the Mechigmen springs are N_2_ and CO_2_; the carbon dioxide content reaches 20–60 vol.% [3]. There are more than 100 individual springs, the majority of which are characterized by a pH close to neutral and temperatures above 60 °C. The hottest springs (up to 97 °C) look like griffons (Figure 2a), surrounded by carbonate-siliceous deposits similar to geyserite. An abundant growth of orange, grey, green, and brownish gelatinous biofilms (Figure 2b) or white/grey and pink filaments (Figure 2c) were observed in these springs. No sulfide odor or yellow–whitish sulfur precipitates were detected.

The Senyavin springs are located in the floodplain of the Klyuchevaya River, 1.5 km from the estuary in the southeast of the Chukotka Peninsula. The sides of the valley formed along the fault zone are composed of granites and gneisses. Springs discharge along the riverbanks on a surface of 500 × 120 m. They emerge from open cracks in the bedrock, low-power alluvial deposits, on the slopes, and in the riverbed. There are more than 150 individual springs (Figure 2d) with temperatures ranging from 20 to 80 °C and a pH of around 8.0. The main evolving gas in these springs is N_2_ (about 98 vol.%) [3]. Grey or black sediments often cover the bottom and sides of springs with temperature over 60 °C and low Eh.

The Chaplino thermal group is located at the Chaplino Cape, 60 km to the east from Provideniye town. Springs discharge at the right bank of the broad Ulchum River floodplain, 5 km above its estuary. The TDS value of these springs is high, up to 20 g/L [2]. The hottest springs (40–80 °C) form two groups at the foot of the high terrace. The first group (named “Upper”) is discharged in a hot creek with a water temperature from 55 to 78 °C and a pH of 6.8–7.5, covered by white–yellow loose sediments and pink microbial flocs (Figure 2e). A well with 87 °C fluid and a few pools from the abandoned recreation center, with water temperatures ranging from 60 to 67 °C and a pH of 7.3, make the second group (named “Lower”). Brown–yellow and grey-green biofilms cover the wooden sides of the pools (Figure 2f).

### 2.2. Water Chemistry Analyses and Mineralogy of the Sampling Sites

For water chemistry analyses, 15 mL Hungate tubes were completely filled with the hydrothermal water and sealed with rubber stoppers and plastic screw caps. The water for control samples was taken from the floodplains of the rivers, where all the hot springs discharge (upstream from the hot springs area). For mineralogical studies, samples of sediments were taken in 50-mL flasks with gas-tight butyl rubber stoppers and aluminum compressible caps. All samples were hermetically sealed, stored, and transported to the laboratory at +4 °C. The temperature, pH, and Eh in the springs were measured in situ. 

Inductively coupled plasma mass spectrometry (ICP-MS) and atomic emission spectroscopy (ICP-AES) analyses were carried out at IPTM RAS (Chernogolovka, Russia) using a Perkin Elmer ELAN model DRC-e mass-spectrometer for the elemental analysis of waters. The atomizing argon flow rate was 0.92–0.95 L/min, the auxiliary flow of argon was 1.17 L/min, and the flow of orifice argon was 15 L/min. The plasma generator capacity was 1270 W, and the detector voltage was 1400 V.

For mineralogical studies, samples were analyzed using X-ray powder diffraction (XRD) approach with a DRON-3M powder diffractometer (Bourevestnik Inc., Moscow, Russia). The dried samples were dispersed in ethanol into powder and applied to a glass cuvette. The X-ray radiation of Co-anode (Co-Kα) was used, with an exposure speed of 4 °C/min. According to the spectrum peaks, the mineral composition was calculated through comparisons with standard samples.

### 2.3. High-Throughput 16S rRNA Gene Amplicon Sequencing

For DNA isolation, samples of sediments, microbial mats, and filamentous biofilms were taken aseptically in 2-mL Eppendorf tubes with screw caps and then fixed with RNAlater™ Stabilization Solution (Thermo Fischer Scientific, Waltham, MA, USA). During transportation and storage, the fixed samples were maintained at +4 °C and then stored at −20 °C until the DNA was extracted. For DNA extraction, the samples were subjected to intensive bead beating with a Minilys™ homogenizer (Bertin Technologies, France), followed by a phenol-chloroform DNA extraction protocol [15]. Prior to bead-beating, the mucous microbial mats were lyzed in a CTAB extraction buffer (2% CTAB, 100 mMTrisHCl, 20 mM EDTA, 1.4 M NaCl, 0.2% β-mercaptoethanol, 0.1 mg/mL proteinase K) for 1 h at 60 °C to get rid of excessive polysaccharides.

To minimize the effect of experimental biases on the biological conclusions, DNA samples were split into two parts and were ran in two different laboratories by two different primer sets and library preparation methods.

V3-V4 hypervariable region libraries were prepared using fusion dual-barcoded primers described by Fadrosh et al. [16], with a slight modification: the rRNA-annealing part of the forward primer corresponded to Pro341F primer, described by Takahashi et al. [17]. PCR amplification of 16S rRNA genes was performed by qPCRmix-HS™ SYBR mastermix (Evrogen, Moscow, Russia) using the following conditions: 30 cycles of denaturation at 95 °C for 15 s; primer annealing at 58 °C for 15 s; and DNA synthesis at 72 °C for 25 s, followed by a final incubation for 5 min at 72 °C. Purification of the PCR products was done using the Cleanup Mini kit (Evrogen, Moscow, Russia). The quality of the final libraries was assessed using electrophoresis in agarose gel. 

V4 amplicon libraries were prepared by a two-stage PCR approach described by Gohl et al. [18]. In the first round of PCR 16S rRNA gene-annealing part of the primers corresponded to 515F [19] and Pro-mod-805R [20] primers. The remaining part of the primers corresponded to the Illumina TruSeq sequencing primer adapter. PCR amplification was performed by qPCRmix-HS™ SYBR mastermix (Evrogen, Moscow, Russia) using the following conditions: 25 cycles of denaturation at 95 °C for 15 s; primer annealing at 56 °C for 20 s; and DNA synthesis at 72 °C for 20 s, followed by a final incubation for 5 min at 72 °C. The second round of PCR was performed with double-indexed primers, which consisted of a TruSeq sequencing primer adapter, 6-bp barcode, and P5 or P7 flow cell-annealing adapter [18]. Amplification was performed by HS Taq DNA-polymeraze (Evrogen, Moscow, Russia) in the following conditions: 14 cycles of denaturation at 95 °C for 20 s, annealing at 59 °C for 20 s, elongation at 72 °C, for 30 s, and a final elongation step at 72 °C for 5 min. The amplicons were purified using the Standard Cleanup Gel Extraction Kit (Evrogen, Moscow, Russia).

All libraries were sequenced with MiSeq™ Personal Sequencing System technology of Illumina Inc. (San Diego, CA, USA) using paired-end 250-bp reads. Demultiplexing was performed with the deML package [21]. After demultiplexing, all reads were subjected to stringent quality filtering, and parts of reads, corresponding to 16S rRNA primers, were removed using CLC Genomics Workbench 10.0 (Qiagen, Stockach, Germany). After the adapter removal, paired reads were merged using the SeqPrep tool (https://github.com/jstjohn/SeqPrep). Finally, 10–65 thousand of merged reads were used for the analysis. The median length of merged reads was 405 and 251 bp for V3–V4 and V4 amplicons, respectively.

### 2.4. Data Analysis

The resulting demultiplexed sequence files for each sample were used as an input for the Mothur v. 1.39.5 16S amplicon analysis package [22]. Due to the fact, that the results of the sequencing of two different amplicons cannot be used for reliable OTU generation, the alpha-diversity analysis was performed separately for each amplicon set. The generation of OTUs was made in Mothur with an OptiClust algorithm [23]. Taxonomic classifications of the resulting OTUs were performed by a Bayesian classification algorithm [24] using the Silva132 database [25]. 

In turn, for the analysis of beta-diversity between different datasets (V3–V4 and V4), both amplicon sets were analyzed by a Mothur phylotype analysis pipeline (https://www.mothur.org/wiki/Phylotype). Visualization of data was performed in an R environment using Phyloseq [26] and ggplot2 [27] packages. Alpha and beta diversity analyses were performed with a Phyloseq package [26].

## 3. Results

More than 50 samples of sediments, microbial mats, filamentous biofilms, minerals, and water were taken from three thermal areas located in the Chukotka Peninsula (Figure 1): Mechigmen (samples 3701–3734), Senyavin (3735–3750), and Chaplino (3751–3754). Water chemistry analyses revealed a high content of alkali metals; W and Tl were present in high concentrations, compared with surrounding waters (Table S1). 

### 3.1. Mineralogy of the Sampling Sites

A characteristic feature of Mechigmen hydrothermal sediments was the presence of a high amount (up to 36%) of pyrite (FeS_2_), as well as X-ray-unidentifiable Fe^3+^-containing amorphous phase—most likely ferrihydrite (ochre deposits covering sides, sediments, and microbial mats; Figure 2a,b)—and secondary minerals (calcite, CaCO_3_; kaolinite, Al_2_Si_2_O_5_(OH)_4_, and several silicates) formed during the transformation of igneous rocks (Table S2). The mineral composition of Senyavin and the “Upper” Chaplino springs sediments were similar to each other and differed from the Mechigmen ones by the complete absence of pyrite and amorphous minerals. On the contrary, a significant predominance of the primary minerals (quartz, potassium feldspar, and plagioclase) and secondary Fe^2+^-containing phyllosilicates of chlorite and hydromicas groups (up to 23%) were detected (Table S2). Sediments collected from the pool of the “Lower” Chaplino thermal group primarily contained X-ray-unidentifiable amorphous minerals, most probably, iron hydroxides, with a small admixture of quartz and biotite. Most likely, amorphous iron minerals either were products of the chemical precipitation from water or originated from microbially driven determined processes that took place in the sediments.

### 3.2. Analysis of Microbial Diversity Using High-Throughput Amplicon Profiling

Twenty-one samples, including 16 samples from the Mechigmen group, four samples from the Senyavin group, and one sample from the Chaplino group, were profiled by high-throughput sequencing of V3-V4 16S rRNA gene amplicons; 12 samples were additionally sequenced with V4 hypervariable region primers. For each sample, 12–67 thousand reads were obtained in each experiment. The total number of OTUs, generated by the OptiClust algorithm with a 3% identity cut-off (which is regarded as a species delineation [28]), was 5376 and 14,617 for V3–V4 and V4 datasets, respectively. Filtering out the OTUs represented by less than 10 sequences in all the samples resulted in 632 and 775 OTUs for the V3-V4 and V4 datasets, respectively. Direct classification of the denoised sequence reads of the mixed dataset by a Mothur phylotype pipeline (https://www.mothur.org/wiki/Phylotype) resulted in the detection of 1308 genera (according to Silva 132 taxonomy) and 687 genera after filtering low abundant (represented by less than five sequencing reads in all dataset) sequences. A similar number of species and genera (632/775 and 687) in the studied samples might be due to low within-genus species diversity or a consequence of differences in OTU-based and direct classification approaches.

**Figure 3:**
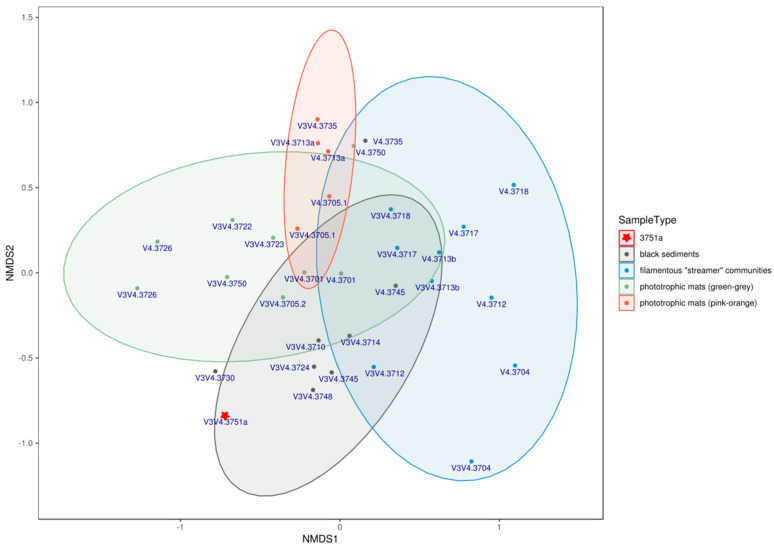
NMDS based on the taxonomic profiles of samples. Ellipse lines, drawn using stat_ellipse function of ggplot2 R package [27] assuming a multivariate t-distribution, are based on the variance observed among each group of samples, except sample 3751a, being a single sample from the Chaplino group. The stress level of analysis returns a value of 0.178, which is considered an acceptable adjustment over the 2D plane. ANOSIM analysis of chosen sample groups showed a significance value of 0.001 and ANOSIM statistic value of 0.4314 which reflects a significant difference between chosen groups.

Rarefaction curves for all datasets showed that the depth of the sequencing was appropriate for most of the samples (Figures S1–S3). Prior to the alpha-diversity analysis, reads were rarefied to the value equal to the minimal number of reads acquired for each dataset. The number of OTUs with >1% abundance varied from 3 to 20 per sample for V3–V4 and from 1 to 16 for the V4 dataset. For the combined dataset, the number of significantly (>1%) abundant genera ranged from 1 to 24 (Table 1). Analysis of the alpha diversity metrics revealed that (i) richness (Chao1 metric) and (ii) quantitative species richness/evenness (Shannon and inverse Simpson indexes) varied significantly between the samples (Table 1). A comparison of diversity metrics obtained for each sample by different methods showed that the influence of primers used for amplification was significantly higher than the choice of analysis method (OTU-based vs. direct classification of sequencing reads). Nonmetric multidimensional simulation (NMDS) analysis of abundance profiles, based on Bray–Curtis distances and ANOSIM analysis of defined groups supported the hypothesis that taxonomic compositions are mainly defined by the attribution of a particular community to one of four major habitat types sampled (Figure 3), with the exception of sample 3751a, which was taken from the “Upper” Chaplino thermal field.

**Figure 4:**
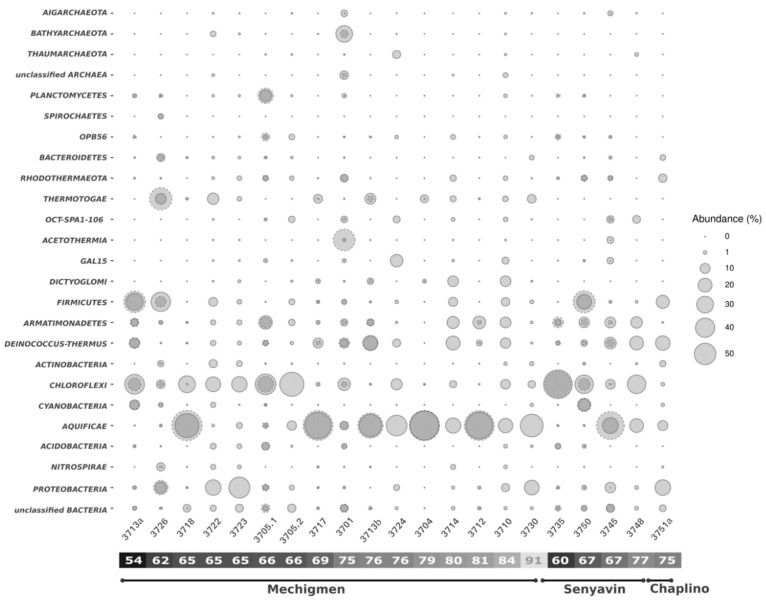
Chukotka hot springs 16S rRNA gene sequence-based microbial abundance analysis at the phylum level. Phyla, with less than 1% of representatives were grouped in “Other”. The relative abundance of phyla in microbial communities is proportional to the circle area. V4 amplicon data represented by circles with dashed borders, while V3–V4 amplicon data by circles with solid borders.

Depending on the sample, 92.3–100% of the 16S rRNA reads were assigned to domain Bacteria, whereas 0–7.7% belonged to domain Archaea. Bacterial 16S rRNA reads were assigned to 25 phyla (each represented by more than 1% of the community in at least one of the samples), and the majority fell into phyla *Aquificae* and *Chloroflexi* (Figure 4). *Aquificae* representatives were found in almost every spring with water temperatures above 65 °C, representing 7–92% of the microbial community. All of the *Aquificae* genera found in the Chukotka springs belonged to the order *Aquificales*, known to contain microaerophilic autotrophs (Figure S4). Among them, the majority of reads belonged to two genera: *Sulfurihydrogenibium* (family *Hydrogenothermaceae*) and *Hydrogenobacter* (family *Aquificaceae*). The only exception was the microbial loose sediments of the “Upper” Chaplino group (sample 3751a) that was characterized by a lower temperature and higher Eh, where *Aquificae* were represented mainly by the genus *Hydrogenivirga* (family *Aquificaceae*) (Figures S4 and S5).

The next most abundant OTUs in the analyzed samples belonged to the genus *Chloroflexus* of the *Chloroflexi* phylum. It dominated in springs with temperatures below 75 °C. Another *Chloroflexi* genus present in CHS was *Thermoflexus* (V3-V4 reads). Uncultured members of this phylum were also detected in noticeable amounts at 65–76 °C (Figures S4 and S5).

Other major phyla were *Proteobacteria* (α- and γ-), *Firmicutes*, *Armatimonadetes* (uncultured), and *Deinococcus-Thermus* (mainly *Thermus* sp.), which were found in almost all samples (Figure 4). In certain springs, *Thermotogae* (mainly *Fervidobacterium*), *Dictyoglomi*, *Cyanobacteria* (mainly *Chlorogloeopsis*), *Rhodothermaeota* (mainly *Rhodothermus*), *Acidobacteria*, *Actinobacteria,* and *Planctomycetes* phyla were also present. Finally, a significant number of sequences belonging to uncultivated phyla, such as candidate divisions Oct-Spa1-106, GAL15, and OPB56 were also detected in some springs (Figure 4, Figures S4 and S5).

Bacteria significantly outnumbered Archaea in the majority of the studied springs (Figure 4), constituting 0–7.7% of all prokaryotes. Sample 3701, where archaea represented up to 40% of all the V3-V4 amplicon reads, made a remarkable exception. However, only 7.7% of V4 amplicon reads in this sample were assigned to Archaea (Figure 4), which might be explained by a systematic underestimation of *Acetothermia* by V3–V4 amplicon reads due to the increased length of the V3 variable region in this phylum and significant amplification biases [29]. Therefore, the V4 amplicon data for that particular sample look more reliable than those obtained for the V3-V4 region. Within domain Archaea, the representatives of *Bathyarchaeota*, *Aigarchaeota*, and *Thaumarchaeota* phyla were also detected. No *Euryarchaeota* or *Crenarchaeota* were found in the CHS samples.

## 4. Discussion

While the hot springs of active volcanic areas strongly varied in pH, chemical content, gas concentration, and so forth, the hydrothermal springs from nonvolcanic areas (e.g., rifts) are mostly similar in physicochemical parameters [30]. In general, the same is correct for CHS, which is studied in this work. However, there was an evident difference in water composition in the springs of distantly located thermal fields. Mechigmen springs were characterized by higher concentrations of Mg and Be and the presence of soluble Fe, while the Mo content was very low. This finding was in agreement with a high content of Fe^2+^-containing minerals found only in the sediments of this group of springs. The water of Senyavin contained lower concentrations of Na, Ca, Mg, K, Li, and Cs compared to the Mechigmen and Chaplino springs. The content of Na, Ca, Br, S, and Sr in the Chaplino hot water was much higher than in the other thermal fields studied, which was in agreement with the highest TDS values and sulfate concentration [3]. However, in general, the chemical composition of CHS was typical for near-neutral nitrogen-chloride thermal springs of non-volcanic origin [30,31].

Five major groups of samples were profiled by high-throughput sequencing of 16S rRNA gene amplicons: two types of microbial mats—orange-pink (Figure 2d) and green-grey-brownish (Figure 2b); filamentous “streamer” communities (Figure 2c); black sediments (Figure 2a); and unsorted samples (Table 1). The fifth group consisted of only one sample (3751a) from a hot creek with white–yellow loose sediments (Figure 2e) taken from the Chaplino thermal field, which differed significantly in the chemical composition of water. The analysis revealed the prevalence of Bacteria (25 phyla) over Archaea (five phyla) in all five groups of samples. NMDS analysis of abundance profiles, as well as ANOSIM analysis of defined groups (Figure 3), confirmed that visual assessment of samples might serve as a reliable indicator of the microbial communities’ similarity.

**Figure 5:**
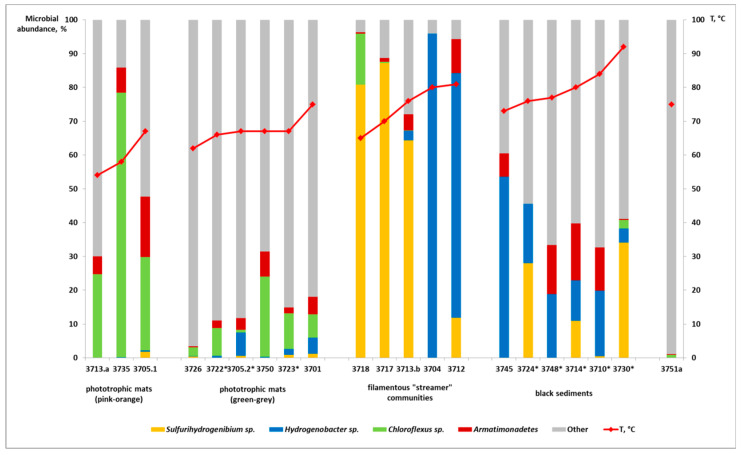
Comparison of the relative abundance of the most numerous taxonomical groups (*Sulfurihydrogenibium* spp., *Hydrogenobacter* spp., *Chloroflexus* spp., and *Armatimonadetes* representatives) in five major CHS habitat types.

Microbial mats, especially orange-pink ones (Figure 2d), were present in almost all the studied thermal fields and were dominated by phototrophic bacteria of genus *Chloroflexus*, reaching up to 80% of the total community (Figure 5). The color of the mats was due to a high amount of *Chloroflexus* representatives. In green–grey–brownish mats, the alpha-diversity (within the sample) metrics was the highest among all five groups of springs (Table 1) and included mainly organoheterotrophic representatives of *Proteobacteria*, *Firmicutes*, *Thermotogae,* and *Chloroflexi* (Figure 4, Figures S4 and S5). Despite the proportion of *Chloroflexus* spp. being much lower there when compared to the pink-orange mats, it was still relatively high (Figure 5).

Typically in hot springs, *Chloroflexus* spp. form microbial mats with *Cyanobacteria* and are believed to act as anaerobic photoheterotrophs in the daytime and aerobic chemoheterotrophs during the night [32]. However, in microbial mats, they could uptake inorganic carbon during the low-light period of the day in the early morning and before sunset when only long-wave light reaches the surface [33,34]. In addition, the study results of *Chloroflexus* pure cultures, as well as genomic analyses, provide evidence of their capability to grow autotrophically [35,36,37]. Taking into account that, besides lower insolation, the sunlight at the polar regions is characterized by a shift of the wavelengths into a longer spectrum [38], it could be assumed that *Chloroflexus* spp. in CHS have advantages, as the absorbance of their bacteriochlorophyll *c* in a long-wavelength range (around 730 nm) [36,39] makes possible their photosynthetic lifestyle during the prolonged polar day. Consequently, *Chloroflexus* representatives seem to play a major role in CHS primary production during the polar day, replacing the cyanobacteria detected in CHS only in trace amounts (less than 1%). In only two mats (samples 3713a and 3750), the cyanobacteria content reached 7–18% (Figure 4, Figures S4 and S5). Effective photolithoautotrophs, cyanobacteria play a major role as primary producers in many environments [40]. The variety of their light-harvesting structures help them to adapt to near-UV to red-light environmental niches [41]. In particular, they acclimate and often become the dominating phototrophs in polar environments [40]. As to the polar hydrothermal springs, while *Cyanobacteria* were dominant in the hot springs with high sulfide concentrations [10], *Chloroflexi* in low-sulfide hot springs outnumbered them [42]. Yet, it was unclear whether these *Chloroflexi* made a significant contribution to primary production (acting as photosynthetic microorganisms), as several chemosynthetic autotrophs were also present there. Thus, it seems that a combination of low insolation, a shift of light spectrum to the long wavelengths during the polar day, low sulfide content, and a rather high water temperature in CHS makes it possible for *Chloroflexi* to outcompete *Cyanobacteria* and play the role of primary producers in CHS. It should also be mentioned that during the Arctic winter, in continuous darkness, the bacteria of *Chloroflexus* genus might switch their metabolism to aerobic chemoheterotrophy [32], which allows them to thrive on a year-round basis.

In filamentous “streamer” communities, *Aquificae* were the most abundant microorganisms (Figure 4 and Figure 5). They were found in almost all hot springs with temperatures above 65 °C, where they formed grey or colorless biofilms (Figure 2c) and constituted up to 92% of the total microbial communities. Surprisingly, *Aquificae* representatives (from 20–50% of the community) also dominated in Fe-rich black sediments (Figure 2a and Figure 5). The members of *Aquificae* are the most widely distributed and abundant lithoautotrophic bacteria in global high-temperature terrestrial hot springs [43,44,45,46,47,48,49,50,51,52,53]. They assimilate carbon dioxide by using the energy of inorganic electron donors (H_2_, reduced sulfur compounds, Fe^2+^, and AsO_3_^3−^) with various electron acceptors (O_2_, NO_3_^−^, S°, S_2_O_3_^−^, SO_3_^−^, Fe^3+^, AsO_4_^3−^, and SeO_3_^2−^) [46,53]. *Aquificae* in CHS were represented mainly by *Sulfurihydrogenibium* and *Hydrogenobacter* genera (Figure 5, Figures S4 and S5). In thermal habitats of volcanic origin (e.g., Yellowstone National Park, Iceland, Kamchatka, Philippines, and China regions), *Sulfurihydrogenibium* representatives were found to dominate in microbial communities of sulfide- and elemental sulfur-rich hot springs [44,48,54,55,56]. On the contrary, in the hot springs without significant sulfur content, other representatives of *Aquificae*—genera *Thermocrinis* and *Hydrogenobacter* [42,47,48,49,54,57]—were thriving. At the same time, in nonvolcanic geothermal systems (such as the hot springs in Malaysia, Nevada, or the Baikal Rift Zone) detected *Aquificae* were present as a minor component and mainly consisted of *Thermocrinis* representatives [31,43,58], which are facultatively autotrophic microorganisms [59]. The discovery of abundant *Aquificae* growth in nonvolcanic, low-sulfur CHS extends our knowledge on this group’s distribution and ecological role. We suppose that *Aquificae* thriving in CHS use the Fe^2+^ present in Mechigmen hot springs water as the energy source for aerobic growth, as it was previously shown for *S. azorense* [60]. Some representatives of *Hydrogenobacter* may be also able to use ferrous iron as the energy substrate. In black sediments of these springs, they can use Fe^2+^- and S^2−^-containing minerals, such as pyrite (FeS_2_), abundant in the Mechigmen group hot springs (Table S2). This assumption is supported by the absence of *Sulfurihydrogenibium* in the Senyavin and Chaplino hot springs microbial communities, where pyrite was not detected (Table S2). This hypothesis is in accordance with the discovery of a large number of the same family—*Hydrogenothermaceae*—representatives in iron-rich hydrothermal sites [42,53,61], where they were believed to use iron, not H_2_, as an electron donor [61]. Other assumptions on the *Aquificae* ecological function in CHS, such as their active oxidation of dissolved hydrogen and reduced sulfur compounds, or their organotrophic growth supported by the input of exogenous organic matter, could explain such an abundant growth of filamentous *Aquificae* biofilms.

The role of aerobic microorganisms in Fe^2+^-containing minerals oxidation is well studied for acidophiles and much less studied for neutrophiles. Still, there are several reports on the ability of aerobic neutrophilic microorganisms to pyrite oxidation [62,63,64]. Besides pyrite, ferrous iron from secondary layered silicates of hornblende, smectite, chlorite, and hydromicas groups present in sediments of CHS could serve as the electron donor for thermophilic lithotrophs. The capability of lithotrophic aerobic or nitrate-reducing microorganisms to use phyllosilicates as electron donors were previously shown for subsurface environments [65,66]. One of the first isolates obtained from CHS (*Tepidiforma bonchosmolovskayae*), an aerobic thermophilic bacterium from a Chaplino thermal spring, is capable of growing chemolithoautotrophically on Fe^2+^-containing mineral (siderite, FeCO_3_) at 42–60 °C [67]. Other inorganic donors (e.g., hydrogen, soluble sulfur compounds, ammonium, carbon monoxide, or nitrite) could not replace siderite. This supports our idea that Fe^2+^-containing minerals play an important role in the autotrophic part of microbial communities of CHS.

In addition to *Aquificae,* a large number of currently known organoheterotrophic phyla, such as *Deinococcus-Thermus*, *Dictyoglomi*, *Thermotogae,* and *Armatimonadetes,* were observed in black, Fe-rich sediments (Figures S4 and S5). Representatives of phylum *Armatimonadetes* deserve special attention because their 16S rRNA sequences were detected in almost all CHS, sometimes as one of the major components of the microbial community (Figure 5). At present, little is known about this phylum previously designated as OP10 candidate division [68]. So far, all isolated *Armatimonadetes* are aerobic, organoheterotrophic, and mostly mesophilic species [69,70,71,72]. However, in analyzed CHS, the members of the currently known *Armatimonadetes* lineages were scarce, and the phylum was represented by a novel lineage of the class level inhabiting hot springs with temperatures above 67 °C (Figure 5), which is in agreement with previous environmental studies [13,43] in which this group was also detected. Interestingly, OP10 bacteria usually make a minor community component, with abundance below 1%; however, their more significant share was detected in metal-rich sites and in carotenoid-pigmented biofilms commonly found in geothermal springs [73]. This fact correlates perfectly with CHS characteristics (high amounts of Fe-containing minerals and domination by colored *Chloroflexus* in bacterial mats), which may indicate the participation of *Armatimonadetes* in redox metal cycling and/or organic matter degradation in CHS (Figure 5).

Not only cultivated phyla but also a high proportion (up to 15% of the total community in some of the samples studied) of candidate divisions (Oct-Spa1-106, GAL15, and OPB56) were detected in microbial communities of CHS, mainly in black sediments (Figure 4). Currently, not much is known about the representatives of these phyla, except for the assumptions—based on the genomic analysis—that they perform aerobic and anaerobic respiration and, most likely, are heterotrophs [74,75,76]. Remarkably, GAL15 and OPB56 are predominantly found in thermal habitats with circumneutral pH and low sulfide contents [74,75,77], the same as in CHS.

Archaea did not exceed 7.7% of the total prokaryotic community in CHS, except spring 3701, where their portion varied from 7.7–40%, depending on the 16S rRNA gene region sequenced. The temperature and geochemistry of this spring were not significantly different from the other CHS, leaving the question of the unique archaeal abundancy unresolved. Archaea were detected only in green–grey mats and black sediments and were represented exclusively by uncultured taxa primarily belonging to *Bathyarchaeota* and *Aigarchaeota* and, in a much smaller quantity, to *Thaumarchaeota* (Figure 4). According to genomic and physiological evidence, *Bathyarchaeota* can be either autotrophs [78] or heterotrophs participating in organic matter degradation [79,80,81,82]. The *Aigarchaeota* representatives detected in CHS were members of “*Candidatus* Caldiarchaeum subterraneum,” whose metabolic capabilities are also unclear. They were previously detected in habitats with circumneutral pH in terrestrial geothermal systems and were found to be able to respire aerobically or anaerobically [76,83] and, perhaps, operate as autotrophs via the dicarboxylate/4-hydroxybutyrate pathway [76,84]. Lack of characteristic inhabitants of volcanic hydrothermal springs—representatives of *Crenarchaeota* and *Euryarchaeota* [44,47,48,49,50,52,85]—is most probably due to the geochemical composition of CHS fluids, where the common substrates hydrogen and/or reduced sulfur compounds [47,48,50,54,85], were absent or present in very low concentrations.

## 5. Conclusions

The first examination of CHS revealed several unique features of their microbial communities because of geographical location and the chemical composition of water and sediments.

- Photosynthetic microbial communities are dominated by green bacteria of genus *Chloroflexus,* outcompeting thermophilic cyanobacteria due to a combination of physicochemical characteristics of CHS and sunlight insolation, characteristic of this polar region.
- In high-temperature CHS, neither sulfur compounds nor sulfur-metabolizing archaea are present, and the growth of dominating organisms—representatives of phylum *Aquificae*—is supposed to be supported by the oxidation of ferrous iron that is dissolved in the water or present in iron-containing minerals.
- Uncultivated and *Candidatus* bacterial and archaeal lineages are rather abundant in some of the CHS, indicating these environments could serve for future metagenomic and cultivation studies of these lineages, their metabolism, and ecological function reconstruction.

## Figures and Tables

**Table 1:** General characteristics of the Chukotka hot springs and alpha diversity metrics of microbial communities there.

Sample	T, °C	pH	Eh, mV	Coordinates	Number of OTUs Detected	Number of OTUs with More than 1% Abundance	Number of Genera Detected	Number of Genera with More than 1% Abundance	Number of Phyla Detected	Number of Phyla with More than 1% Abundance	Chao1	ACE	Shannon	Simpson	InvSimpson
3713.a	54	7.3	−320	N65.806362/W173.39634	764	10	65	8	16	7	3420.05 ± 402.09	3389.21 ± 34.21	2.47	0.85	6.69
3735	60	8.0	+210	N64.73667/W172.84869	376	6	35	6	18	4	1556.17 ± 240.7	2034.59 ± 24.84	1.16	0.36	1.57
3705.1	66	7.0	ND	N65.806419/W173.39557	1843	13	148	12	29	11	7801.98 ± 571.02	8676.04 ± 58.47	2.88	0.88	8.65
3726	62	6.8	−400	N65.805696 W173.39655	1116	15	377	18	28	11	2398.71 ± 156.76	2217.86 ± 28.81	4.06	0.94	16.71
3722	65	6.7	−100	N65.805433/W173.39929	860	17	168	26	34	12	4058.07 ± 554.57	3321.55 ± 36.41	4.08	0.96	22.37
3705.2	66	7.0	−140/−200	N65.806419/W173.39557	1027	11	147	9	32	9	3688.38 ± 353.27	3478.97 ± 37.97	2.27	0.66	2.94
3750	67	8.0	−300	N64.736091/W172.84917	705	10	169	10	25	9	2568.96 ± 312.22	2122.26 ± 29.01	2.43	0.81	5.31
3723	65	6.7	−70	N65.80555/W173.39721	1090	17	199	17	36	12	3590.43 ± 307.57	3679.47 ± 40.28	2.97	0.81	5.39
3701	75	6.8	−120/−20	N65.806401/W173.39534	503	20	147	17	39	15	1710.71 ± 219.36	1790.2 ± 27.12	3.41	0.93	15.06
3718	65	6.8	−326	N65.805704/W173.40537	729	3	85	3	22	3	3149.37 ± 360.19	3513.99 ± 34.05	1.19	0.53	2.13
3717	69	6.8	−326	N65.805566/W173.40547	878	6	129	5	29	6	3188.73 ± 305.45	3526.48 ± 37.68	1.15	0.38	1.61
3713.b	76	6.7	−300	N65.806362/W173.39634	473	8	42	5	20	5	1991.07 ± 279.21	2166.89 ± 27.29	1.87	0.68	3.15
3704	80	6.7	−278	N65.806329/W173.39563	90	3	28	3	14	3	275.77 ± 68.09	311.17 ± 4.61	0.4	0.15	1.18
3712	81	6.4	−305	N65.806335/W173.39644	1109	4	115	4	26	3	4077.9 ± 346.39	4632.53 ± 44.21	1.4	0.6	2.51
3745	72	8.1	−250/−50	N64.73656/W172.8518	649	15	56	13	17	12	4526.68 ± 741.86	3924.76 ± 25.81	2.89	0.89	8.87
3724	76	6.7	−96	N65.805774/W173.397	446	12	77	11	25	9	1375.96 ± 176.55	1434.89 ± 24.48	2.71	0.88	8.05
3748	77	8.0	−310	N64.73644/W172.85123	401	7	87	7	20	7	1148.64 ± 147.97	1089.69 ± 20.16	1.93	0.78	4.62
3714	79	6.7	−316	N65.806362/W173.39634	854	16	69	12	25	11	3683.68 ± 396.69	4252.5 ± 41.12	2.86	0.92	12.05
3710	84	7.0	−40	N65.806271/W173.39639	562	14	70	16	28	15	2533.27 ± 354.46	3666.54 ± 30.06	3.25	0.92	12.08
3730	94	6.9	−360	N65.805844/W173.39485	403	8	148	9	23	7	1964 ± 433.64	1511.87 ± 24.28	2.83	0.81	5.4
3751a	75	7.5	+30/+80	N64.435798/W172.52126	709	18	135	17	19	10	4378.12 ± 704.19	3836.49 ± 34.84	3.77	0.94	15.39

The five major habitat types sampled include pink-orange microbial mats (orange), green-grey microbial mats (green), filamentous “streamer” communities (blue), black Fe-rich sediments (grey), and unsorted hot creek with white-yellow loose sediments is highlighted by a yellow color. ND—not detected.

## Supplementary Materials

The following are available online at https://www.mdpi.com/2076-2607/8/9/1308/s1, Table S1: Chemical composition of thermal waters in Chukotka hot springs, obtained by ICP-MS and ICP-AES analysis, Mineralogy of the Sampling Sites, Table S2: Crystalline mineral composition of the bottom deposits of hot springs studied obtained by XRD analysis, Figure S1: Rarefraction curve of combined V4 and V3–V4 dataset, analyzed by Mothur phylotype pipeline, Figure S2: Rarefraction curve of V3-V4 dataset, Figure S3: Rarefraction curve of V4 dataset, Figure S4: Heatmap reflecting the distribution of 50 most abundant genera in the V3–V4 dataset, Figure S5: Heatmap reflecting the distribution of 50 most abundant genera in the V4 dataset.
