# Supplementary material for: Hot in Cold: Microbial Life in the Hottest Springs in Permafrost
Source: Microorganisms. 2020 Aug 27;8(9):1308. doi: 10.3390/microorganisms8091308 (PMC7565842; doi:10.3390/microorganisms8091308)
Supplement: Supplementary file 1 [file microorganisms-08-01308-s001.pdf]

Tatiana V. Kochetkova, Stepan V. Toshchakov, Kseniya S. Zayulina, Alexander G. Elcheninov, Daria G. Zavarzina, Vasiliy Yu. Lavrushin, Elizaveta A. Bonch-Osmolovskaya and Ilya V. Kublanov

Hot in cold: microbial life in the hottest springs in permafrost

Supplementary Materials

Table S1 Chemical composition of thermal waters in Chukotka hot springs, obtained by ICP-MS and ICP-AES analysis

Element	Mechigmen hot springs					Senyavin hot springs					Chaplino hot springs	
	Control	3701	3710	3728	3730	Control	3735	3737	3745	3747	3751a	3755 (the well)
	C, ppm											
B	0.03	0.68	0.70	0.68	0.70	0.002	0.06	0.06	0.05	0.05	1.59	1.54
Na	31.18	1289.82	1260.23	1313.15	1399.10	2.26	365.34	361.82	328.10	320.87	4396.32	4310.69
Mg	0.72	2.07	2.00	1.75	1.90	0.78	0.28	0.26	0.11	0.09	1.29	1.20
Al	0.03	0.12	0.35	0.08	0.12	0.01	0.08	0.02	0.13	0.04	0.16	0.21
Si	3.45	61.35	59.69	66.12	66.33	1.77	31.78	30.58	27.82	27.22	32.56	34.04
S	4.09	24.92	26.59	24.47	25.37	3.10	20.66	19.32	19.10	19.65	67.77	68.68
K	2.91	87.29	88.69	88.74	95.06	0.38	11.56	11.65	11.28	10.97	120.06	128.27
Ca	7.21	122.58	136.39	129.83	125.92	7.90	101.09	98.00	71.63	72.43	2898.03	2893.06
Mn	0.002	0.17	0.22	0.14	0.16	0.001	0.13	0.02	0.11	0.11	0.10	0.09
Fe	0.07	0.26	3.89	1.87	0.63	0.05	0.09	< LOD	< LOD	< LOD	< LOD	0.22
Zn	< LOD	0.07	0.01	0.01	0.01	0.001	0.01	< LOD	< LOD	0.01	0.02	0.06
Ga	< LOD	< LOD	0.005	< LOD	< LOD	< LOD	0.002	0.002	0.004	0.004	< LOD	< LOD
Ge	< LOD	0.02	0.02	0.02	0.02	< LOD	0.004	0.004	0.003	0.004	< LOD	< LOD
As	< LOD	0.002	0.002	< LOD	0.01	< LOD	< LOD	< LOD	< LOD	< LOD	< LOD	< LOD
Br	0.18	5.42	5.50	5.50	5.57	0.01	2.32	2.38	2.22	2.25	33.21	34.47
Sr	0.19	5.36	5.33	5.64	5.85	0.04	2.32	2.27	2.10	2.06	45.04	45.28
Ba	0.01	0.43	0.60	0.51	0.50	0.003	0.03	0.02	0.02	0.02	0.92	1.15
Pb	< LOD	0.004	0.001	< LOD	0.001	< LOD	< LOD	< LOD	< LOD	< LOD	0.002	0.04
Li	0.10	4.84	4.50	4.97	5.13	0.001	0.47	0.47	0.44	0.43	2.71	2.77

	C, ppb											
Be	0.01	1.57	3.63	3.48	1.78	< LOD	0.39	0.20	0.35	0.42	< LOD	< LOD
Rb	18.69	696.96	732.09	703.56	676.98	1.02	141.09	141.25	128.27	126.16	839.85	868.69
Y	0.13	0.04	15.13	0.40	0.16	0.04	0.05	< LOD	0.14	0.05	< LOD	< LOD
Zr	0.03	0.23	0.30	0.05	0.08	0.003	0.39	0.23	0.30	0.21	< LOD	< LOD
Mo	0.18	0.54	0.29	0.40	0.35	0.61	20.62	21.84	21.28	20.05	15.55	21.16
Sn	< LOD	0.49	0.37	0.38	0.38	< LOD	< LOD	< LOD	< LOD	< LOD	< LOD	< LOD
Sb	0.03	0.44	0.72	0.20	0.56	< LOD	0.03	< LOD	< LOD	0.06	0.89	5.17
Cs	5.87	317.70	326.60	325.50	294.80	0.05	59.60	58.85	53.05	51.75	373.91	398.43
La	0.12	0.35	1.55	< LOD	0.45	0.06	0.35	< LOD	0.15	< LOD	0.12	0.17
Ce	0.14	0.60	3.30	0.20	1.06	0.04	0.62	< LOD	0.35	< LOD	0.20	0.16
Pr	0.03	0.04	0.42	0.02	0.16	0.01	0.01	< LOD	0.04	< LOD	< LOD	< LOD
Nd	0.15	0.37	1.96	0.08	0.54	0.04	0.35	< LOD	0.14	< LOD	< LOD	0.19
Sm	0.03	0.06	0.62	0.03	0.07	0.01	0.11	< LOD	0.03	< LOD	< LOD	1.20
Eu	0.01	< LOD	0.31	< LOD	< LOD	< LOD	0.01	< LOD	< LOD	< LOD	< LOD	0.23
Gd	0.03	0.04	1.16	0.06	0.08	0.01	0.09	< LOD	0.04	< LOD	< LOD	0.54
Tb	0.005	0.01	0.26	0.01	0.01	0.001	0.02	< LOD	0.01	< LOD	< LOD	0.01
Dy	0.03	0.03	1.88	0.05	0.06	0.01	0.09	< LOD	0.02	< LOD	< LOD	< LOD
Ho	0.005	0.004	0.32	0.02	0.01	0.002	0.02	< LOD	0.004	< LOD	< LOD	< LOD
Er	0.01	0.03	0.84	0.04	0.03	0.005	0.06	< LOD	0.02	< LOD	< LOD	0.02
Tm	0.002	0.002	0.11	< LOD	0.004	0.001	0.005	< LOD	0.002	< LOD	< LOD	< LOD
Yb	0.01	0.02	0.61	0.03	0.01	0.004	0.04	< LOD	0.01	< LOD	< LOD	< LOD
Lu	0.002	< LOD	0.08	0.003	0.002	0.001	0.006	< LOD	< LOD	< LOD	< LOD	< LOD
Hf	< LOD	0.02	0.02	0.01	< LOD	< LOD	0.02	< LOD	< LOD	< LOD	< LOD	0.06
W	0.09	22.52	20.90	26.73	19.11	0.06	27.63	28.85	26.03	25.72	15.91	16.38
Tl	0.03	2.76	11.90	3.05	3.36	0.001	0.30	0.27	0.27	0.32	3.83	4.04
Bi	< LOD	0.04	0.02	< LOD	0.01	< LOD	0.03	< LOD	< LOD	< LOD	0.42	4.19
Th	0.01	0.02	0.22	0.02	0.03	0.002	0.04	0.01	0.05	0.01	< LOD	< LOD
U	0.02	0.03	0.03	0.01	0.02	0.17	0.05	0.01	0.02	0.02	< LOD	< LOD

LOD – a limit of detection (Fe=0.008 ppm, Zn=0.003 ppm, Ga=0.0002 ppm, Ge=0.0003 ppm, As=0.0004 ppm, Pb=0.0001 ppm; Be=0.021 ppb; Y=0.01 ppb, Zr=0.01 ppb, Sn=0.19 ppb, Sb=0.014 ppb, La=0.042 ppb, Ce=0.018 ppb, Pr=0.006, Nd=0.011, Sm=0.004 ppb, Eu=0.004 ppb, Gd=0.004 ppb, Tb=0.001 ppb, Dy=0.003 ppb, Ho=0.002 ppb, Er=0.003 ppb, Tm=0.001 ppb, Yb=0.002 ppb, Lu=0.001 ppb, Hf=0.006 ppb, Bi=0.005 ppb, Th=0.002 ppb, U=0.004 ppb);
Control – a water sample from a river, taken upstream to the hot springs area.

Mineralogy of the Sampling Sites

The presence of pyrite, X-ray unknown amorphous phase (ochre deposits covering sides, sediments and microbial mats) (Fig. 2a, d), and secondary minerals formed during the transformation of igneous rocks was a characteristic feature for the sediments of the Mechigmen hydrothermal springs. Almost all springs in the Mechigmen thermal group contained characteristic X-ray amorphous ochre deposits covering sides, sediments and microbial mats in the springs what is most likely due to high content of oxidized Fe-compounds there. The black layer, observed under the ochre layer in sediments and mats, most probably, consisted of the reduced iron compounds (Fig. 2a, d) as when dried on the air its color changed from dark-grey to ochre. The minerals composition of the investigated bottom sediments (Table S2) (samples 3709, 3710, 3714, 3717 and 3730) revealed minerals typical for the enclosing granites and porphyries - quartz, potassium feldspar, plagioclase as well as hydromicas, smectites, mixed mica-smectites and clay minerals. The latter, such as kaolinite, formed as the result of hydrothermal transformation of the minerals of the igneous rocks, primarily micas. Their content fluctuated from 2 to 35% of total minerals content. In 4 of 5 samples (3709, 3710, 3714 and 3730), pyrite (from 9 to 36%) and calcite (from 5 to 21%) were present. The origin of both minerals is most likely due to hydrothermal processes. The presence of gypsum in the sediments of springs 3710 and 3714 may indicate a possible connection between springs of the Mechigmen group and sea water. Sediments of springs 3709, 3710 and 3714 contained X-ray unidentifiable amorphous minerals of 8 to 40%, which, judging by the color of the sediments largely consists of iron hydroxides. The homogeneous composition of the sediments of the investigated hot springs within one group, allow to suggest that the presence of pyrite, X-ray unknown amorphous phase, and secondary minerals formed during the transformation of igneous rocks is a characteristic feature for the sediments of the Mechigmen hydrothermal springs.

Mineralogical composition of the bottom sediments of the Senyavin hydrothermal group (samples 3737, 3745 and 3748) and sample 3751a from the "Upper" Chaplino group was very similar to each other and rather different from the Mechigmen ones. The complete absence of the X-ray unidentifiable amorphous minerals with a significant predominance of the primary minerals quartz, potassium feldspar and plagioclase in the sediments (total content 77, 86, 96 and 78%, respectively) was found. As a secondary mineral in the sediments of both investigated springs, chlorite (4, 5, 2 and 3%, respectively) was present - a phyllosilicate with a high content of ferrous iron (Table S2). Sediments collected from the pool from the "Lower" Chaplino thermal group (sample 3757) contained mainly X-ray amorphous minerals, most probably iron hydroxides, with a small admixture of quartz and biotite. Most likely X-ray amorphous minerals here is a product of chemical precipitation from water, as well as of microbiological processes taking place in artificial pool.

Table S2 Crystalline mineral composition of the bottom deposits of hot springs studied obtained by XRD analysis

Mineral	Mechigmen group					Senyavin group			Chaplino group	
	3709	3710	3714	3717	3730	3737	3745	3748	3751 a	3757
Quartz SiO_2	36	17	28	42	5	38	53	52	44	+
Plagioclase $Na,Ca[AlSi_3O_8]$	8	14	14	21	18	24	24	28	19	-
Potassium feldspar $K[AlSi_3O_8]$	0	0	5	0	8	15	9	16	15	-
Hornblende $(Ca,Na)_{2-3}(Mg,Fe,Al)_5(Al,Si)_8O_{22}(OH,F)_2$	0	0	0	0	0	2	0	0	0	+
Smectite $M_{0.33}, H_2OAl_{1.67}(Fe^{2+}, Mg^{2+})_{0.33} Si_4O_{10}(OH)_2$	0	0	0	0	18	5	0	0	1	-
Chlorite $(Fe^{+2},Mg,Al,Fe^{+3})_6(Si,Al)_4O_{10}(OH,O)_8$	0	0	0	0	0	4	5	2	3	-
Hydromicas $(K,H_3O)(Al,Mg,Fe)_2(Si,Al)_4O_{10}[(OH)_2,(H_2O)]$	0	19	11	5	0	9	5	2	15	-
Mixed mica-smectites	2	6	12	3	0	3	4	0	1	-
Kaolinite $Al_2Si_2O_5(OH)_4$	2	0	0	8	0	0	0	0	2	-
**Pyrite FeS_2**	**36**	**9**	**24**	**0**	**30**	**0**	**0**	**0**	**0**	**-**
Gypsum $CaSO_4 \cdot 2H_2O$	0	8	6	0	0	0	0	0	0	-
Calcite $CaCO_3$	0	0	0	21	21	0	0	0	0	-
Aragonite $CaCO_3$	0	9	0	0	0	0	0	0	0	-
**Amorphous phase $Fe(OH)_3$**	**16**	**8**	**0**	++	**0**	**0**	**0**	**0**	**0**	+++

(++, +++) – high content of unknown amorphous phase in the sample. In this case, the numbers reflect only the ratio of minerals within the crystalline phase.

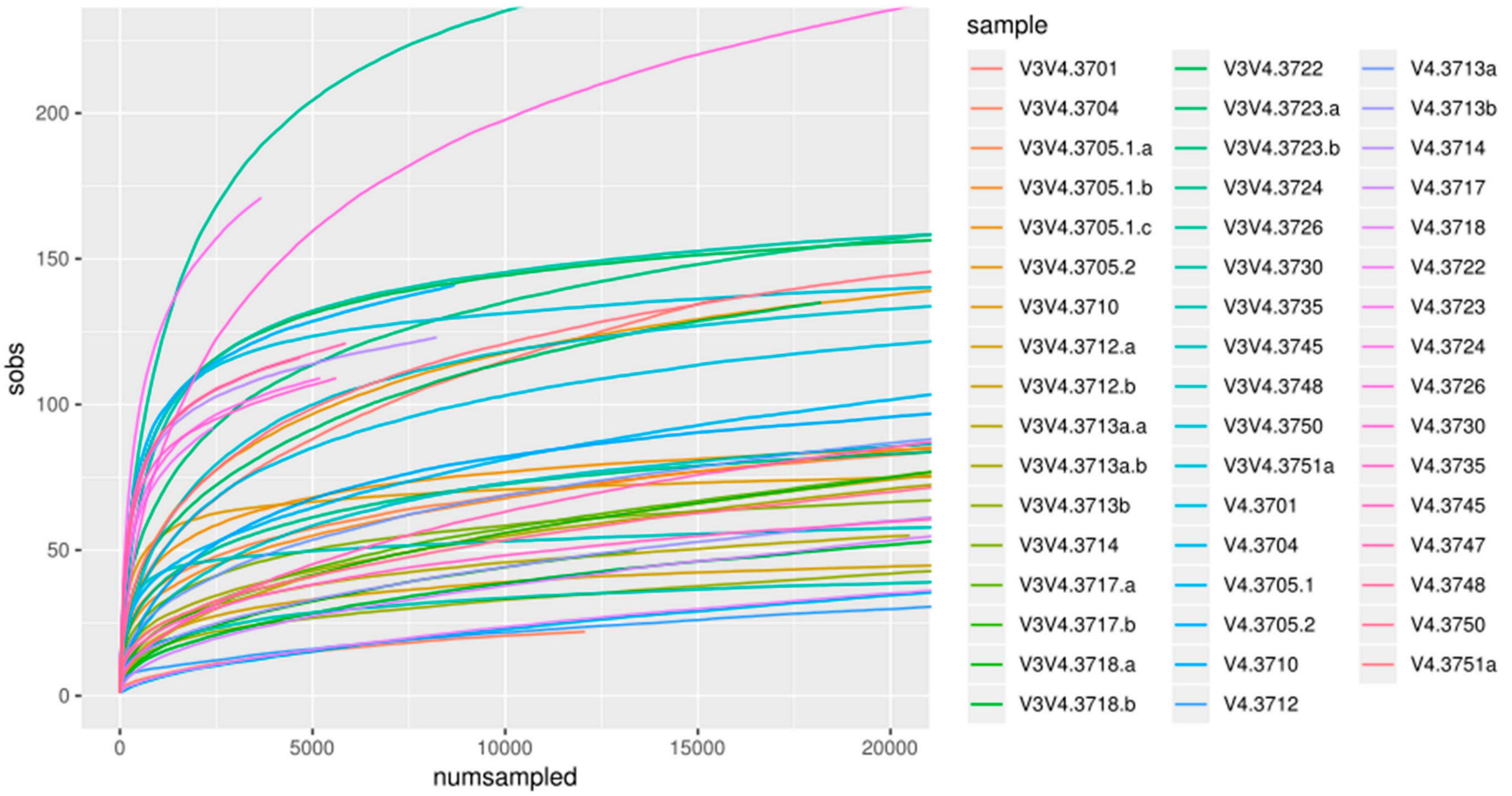

Fig. S1 Rarefraction curve of combined V4 and V3V4 dataset, analyzed by Mothur phylotype pipeline

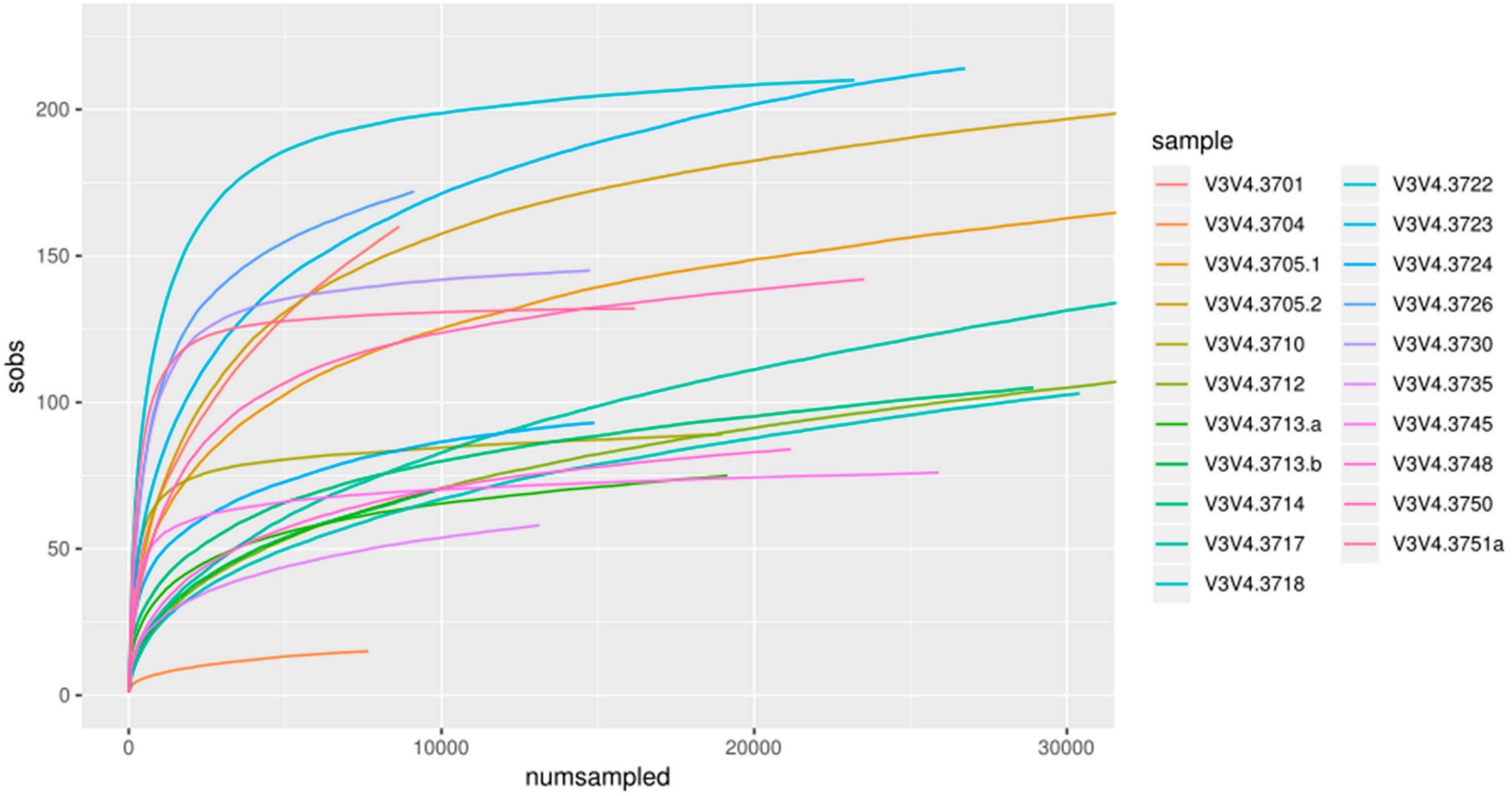

Fig. S2 Rarefraction curve of V3V4 dataset

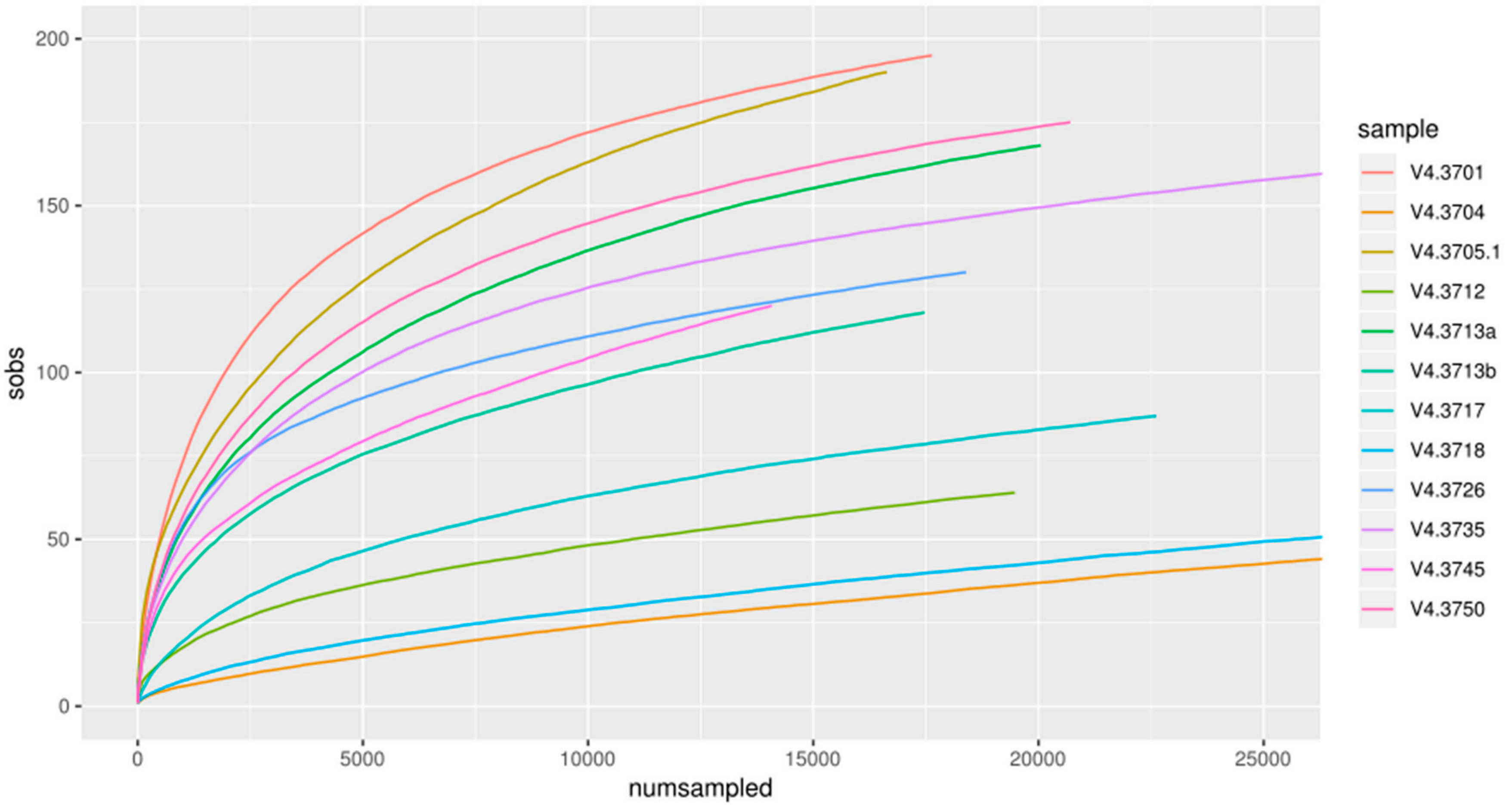

Fig. S3 Rarefraction curve of V4 dataset

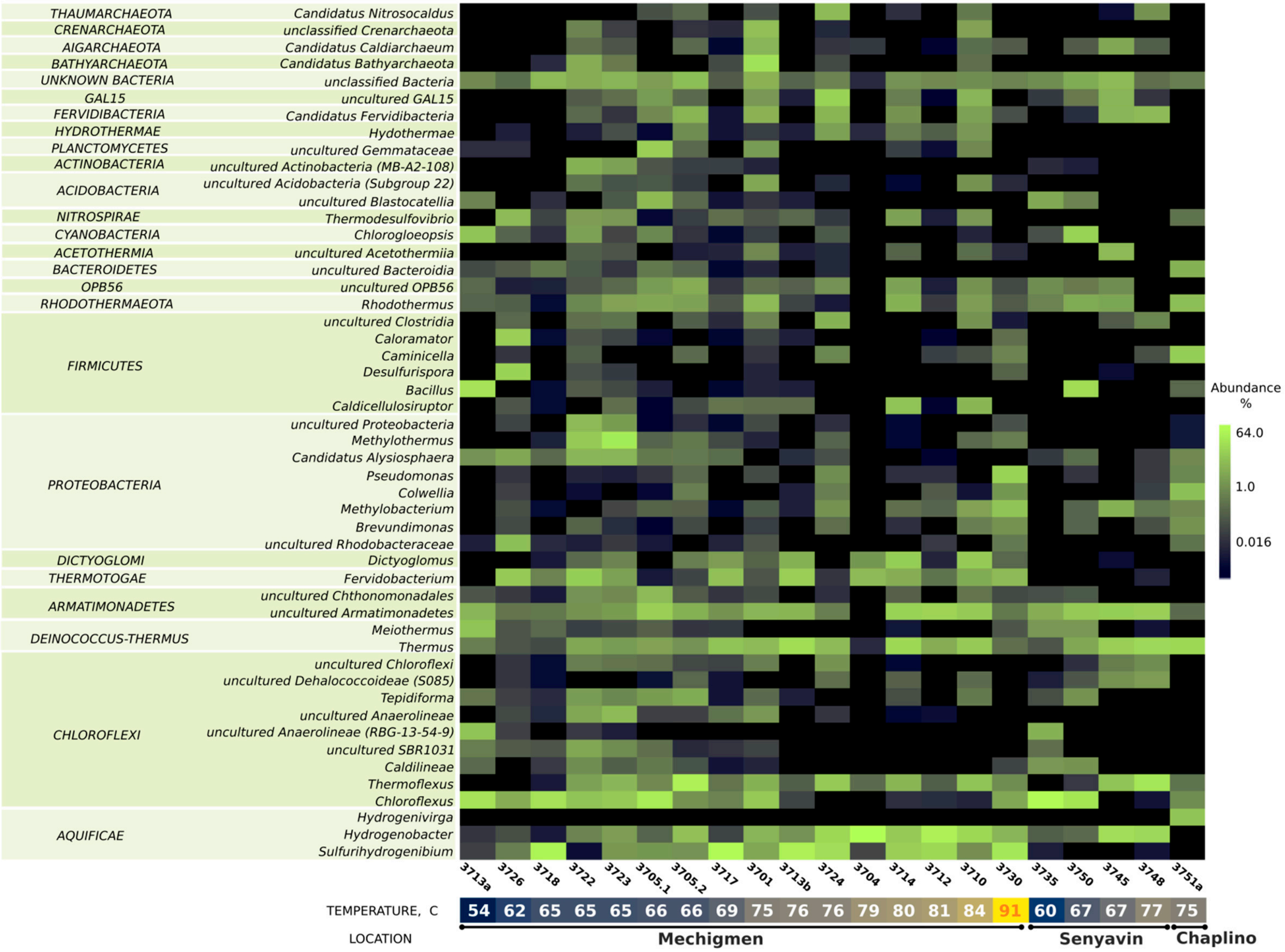

Fig. S4 Heatmap reflecting the distribution of 50 most abundant genera in the V3V4 dataset

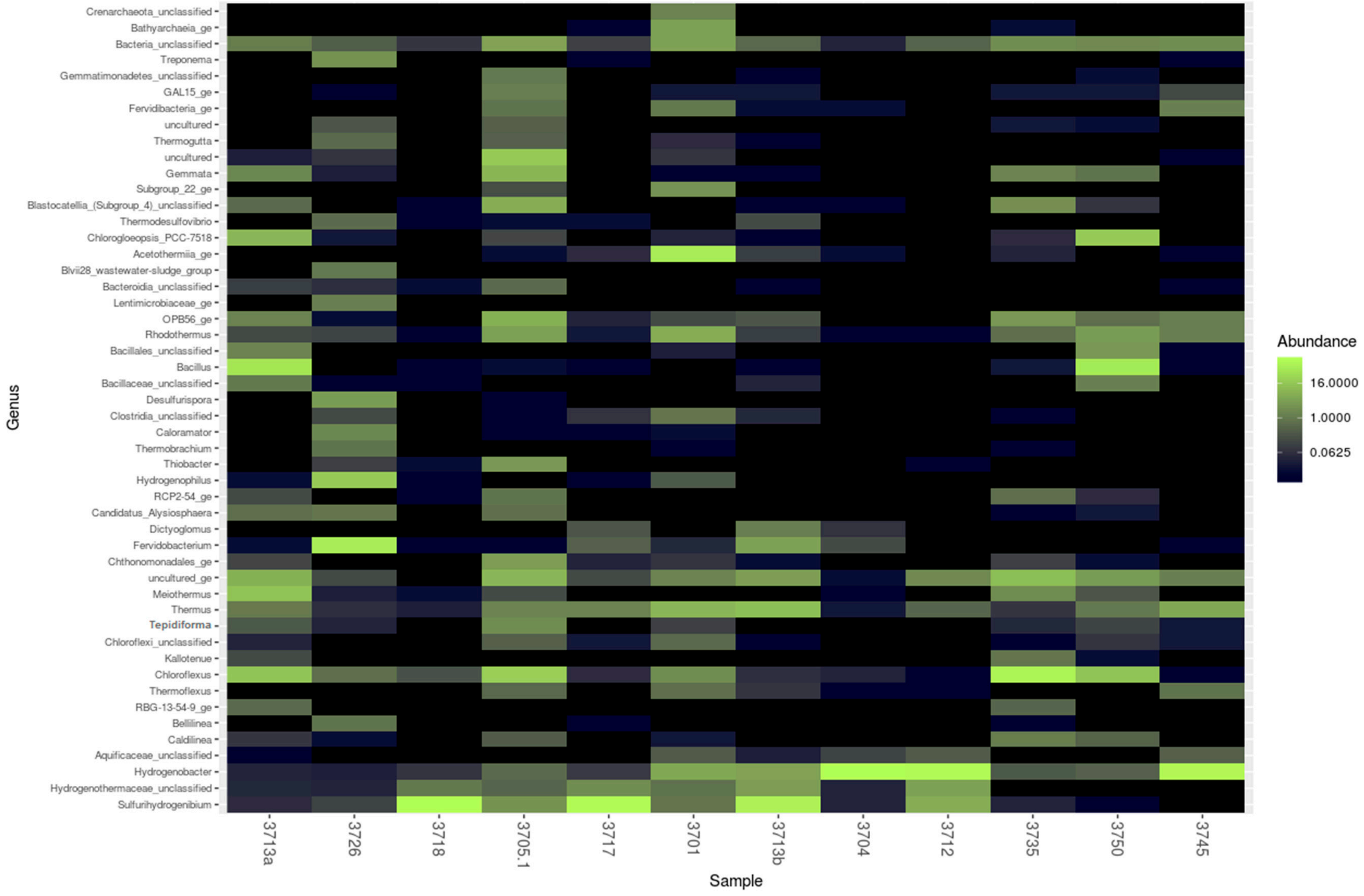

Fig. S5 Heatmap reflecting the distribution of 50 most abundant genera in the V4 dataset
